# When cancer drug resistance meets metabolomics (bulk, single-cell and/or spatial): Progress, potential, and perspective

**DOI:** 10.3389/fonc.2022.1054233

**Published:** 2023-01-06

**Authors:** Zhiqiang Zhang, Chaohui Bao, Lu Jiang, Shan Wang, Kankan Wang, Chang Lu, Hai Fang

**Affiliations:** ^1^ Shanghai Institute of Hematology, State Key Laboratory of Medical Genomics, National Research Center for Translational Medicine at Shanghai, Ruijin Hospital, Shanghai Jiao Tong University School of Medicine, Shanghai, China; ^2^ School of Life Sciences and Biotechnology, Shanghai Jiao Tong University, Shanghai, China; ^3^ MRC London Institute of Medical Sciences, Imperial College London, London, United Kingdom

**Keywords:** cancer drug resistance, metabolic reprogramming, metabolomics, single-cell metabolomics, spatial metabolomics

## Abstract

Resistance to drug treatment is a critical barrier in cancer therapy. There is an unmet need to explore cancer hallmarks that can be targeted to overcome this resistance for therapeutic gain. Over time, metabolic reprogramming has been recognised as one hallmark that can be used to prevent therapeutic resistance. With the advent of metabolomics, targeting metabolic alterations in cancer cells and host patients represents an emerging therapeutic strategy for overcoming cancer drug resistance. Driven by technological and methodological advances in mass spectrometry imaging, spatial metabolomics involves the profiling of all the metabolites (metabolomics) so that the spatial information is captured *bona fide* within the sample. Spatial metabolomics offers an opportunity to demonstrate the drug-resistant tumor profile with metabolic heterogeneity, and also poses a data-mining challenge to reveal meaningful insights from high-dimensional spatial information. In this review, we discuss the latest progress, with the focus on currently available bulk, single-cell and spatial metabolomics technologies and their successful applications in pre-clinical and translational studies on cancer drug resistance. We provide a summary of metabolic mechanisms underlying cancer drug resistance from different aspects; these include the Warburg effect, altered amino acid/lipid/drug metabolism, generation of drug-resistant cancer stem cells, and immunosuppressive metabolism. Furthermore, we propose solutions describing how to overcome cancer drug resistance; these include early detection during cancer initiation, monitoring of clinical drug response, novel anticancer drug and target metabolism, immunotherapy, and the emergence of spatial metabolomics. We conclude by describing the perspectives on how spatial omics approaches (integrating spatial metabolomics) could be further developed to improve the management of drug resistance in cancer patients.

## 1 Introduction

Cancer drug resistance occurs when the tumor is getting insensitive to drug treatment, which also explains tumor recurrence and metastasis (1). Resistance to anticancer drugs may be attributable to a number of factors, including but not limited to: genetic mutations (2–5), epigenetic changes (6–10), drug efflux (11–13), altered target engagements (4, 14), and cellular mechanisms (15, 16).

**Figure 1:**
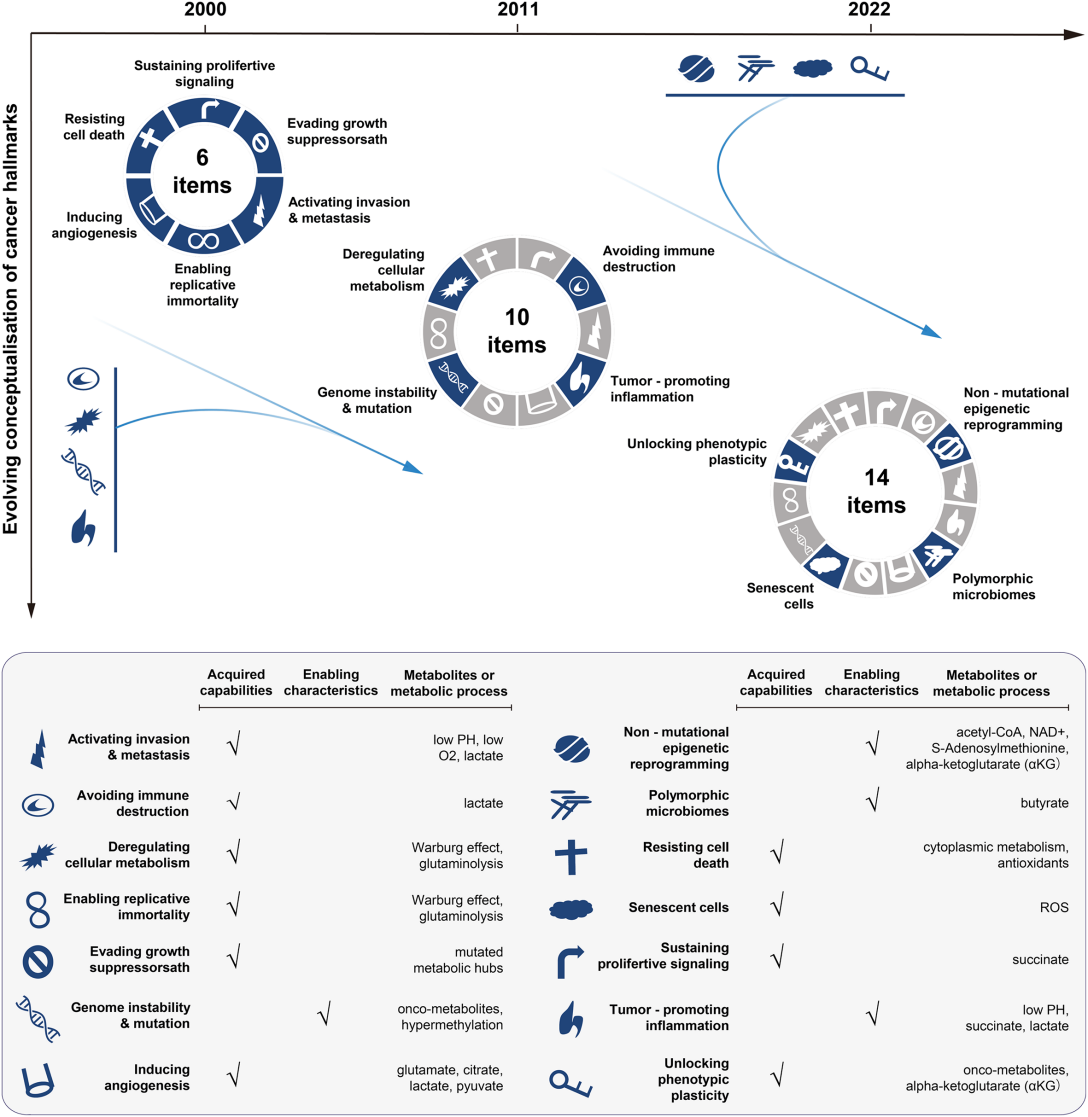
Conceptualisation of cancer hallmarks evolving from 2000 to 2022. The first six cancer hallmarks were presented in 2000. Additional four hallmarks (the metabolism, immunity, inflammation, and genome instability) were added in 2011, with the focus on the metabolism dysfunction in cancer. In 2022, the cancer hallmarks were extended to 14 items, with newly added four hallmarks including: unlocking phenotypic plasticity, senescent cells, non-mutational epigenetic reprogramming, and polymorphic microbiomes. The bottom panel lists the hallmarks and the corresponding metabolic processes or metabolites involved. The illustration is mainly inspired by these three articles (18–20).

The complexity of mechanisms underlying cancer drug resistance requires omics-driven systems approaches. Our focus has long been on mapping the alterations at the genome, epigenome and proteome levels, even though changes at the metabolome level are more closely related to the drug resistance phenotype (17). However, our understanding of metabolic dysfunction remains very limited. Over time, metabolic reprogramming has been recognised as a hallmark of cancer (18–20) (**Figure 1**). Metabolic changes can provide cancer cells with an advantage over normal counterparts in terms of exploiting energy. Furthermore, the altered metabolism can generate a significant amount of intermediate metabolites, which are essential for the biosynthesis of macromolecules and can potentially facilitate cancer proliferation, metastasis, and drug resistance as well (21–24).

Metabolomics, particularly the emerging spatial metabolomics driven by developments in mass spectrometry imaging (MSI) technologies, holds great promise for an improved understanding of cancer drug resistance. As its name suggests, spatial metabolomics can globally profile metabolites, lipids, drugs, and other small molecules; profiled so in the spatial context of cells, tissues, organs, and even the whole organism (25). It is well-suited to generate metabolomic profiles for heterogenous and complex biological systems, such as tumors, where the spatial information about the cancer cells and the tumor microenvironments is captured *bona fide* (26). *Via* spatial metabolomics, metabolic profiles specific to R-CHOP-resistant diffuse large B-cell lymphoma (DLBCL) have been characterised (27), and metabolic enzymes/pathways in esophageal squamous cell carcinoma have been discovered (28). Moreover, metabolomics-led subtypes of gastric cancer patients have been identified to correlate with trastuzumab therapy efficiency (i.e., trastuzumab-sensitive versus trastuzumab-resistant) (29). Recently, spatial metabolomics has been found to be helpful in classifying non-small cell lung cancer (NSCLC) patients into responders and non-responders of neoadjuvant chemotherapy (30).

In the remaining sections of this review, we provide an in-depth appraisal of the technologies and methods currently available for bulk, single-cell, and/or spatial metabolomics, and discuss how these latest advances have improved our understanding of mechanisms underlying the metabolic reprogramming in tumor responses to anticancer drugs. We will also discuss the outstanding challenges involved and share our perspectives on further developing integrative spatial omics approaches to maximise the potential of metabolomics in dissecting cancer drug resistance.

## 2 Advances in developing metabolomics technologies and data-mining tools

Metabolites are essential components of the complex biological system, which reflect the environment in which the cells are located. They are tightly correlated with the effects of drugs, the nutritional status of cells, and the influence of other external factors. The term “metabolomics” describes the global identification and quantification of metabolites. Technologies used to identify and quantify metabolites globally have evolved from classic bulk metabolomics, single-cell metabolomics and spatial metabolomics, and to the emerging spatial single-cell metabolomics.

### 2.1 Bulk metabolomics

**Figure 2:**
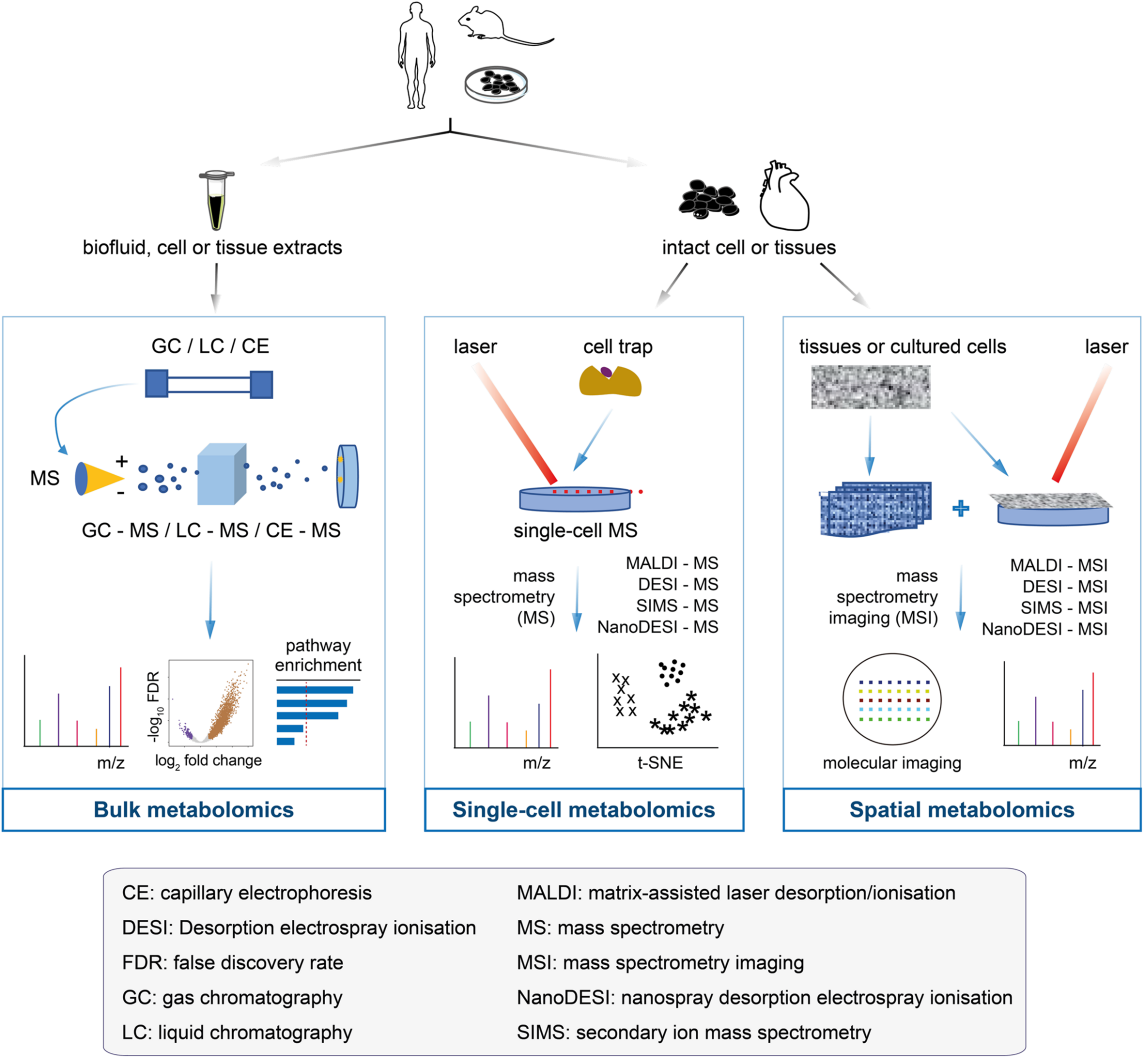
Flowcharts of bulk metabolomics, single-cell metabolomics, and spatial metabolomics. Bulk metabolomics (left panel): the samples are a mixture of biofluid, cell and tissue extracts, with all the molecules unable to get back to the original of primary cells after chromatographic separation. The molecular fragments are ionised in mass spectrometry to measure the m/z profiles and estimate their identity. Traditional analyses, such as differential analysis and pathway enrichment, can be performed. Single-cell metabolomics (middle panel): the cultured cells or tissues are separated into single cells by cell trap, followed by the metabolites of each cell measured by mass spectrometry. The single-cell data make it easier to determine the cell heterogeneity, which is useful for cancer drug-resistant research. Spatial metabolomics (right panel): an emerging field of omics research that has enabled the localisation of metabolites, lipids, and drugs in tissue sections. Spatial metabolomics and its enabling technology (that is, mass spectrometry imaging) generate hyperspectral imaging data that not only receive the m/z profiles, but also provide the access to the locations of the molecules in the cells or tissues for mass spectrometry imaging analysis. Technologies and methods can be used to acquire raw data, thus providing the starting point for computational analysis.

Bulk metabolomics involves the detection and quantification of all metabolites altogether from samples, such as *in vitro* cultured cells, tissues, and biofluids (31–33). Analytical platforms for bulk metabolomics have been diversified over the past decade and mainly include nuclear magnetic resonance (NMR) and mass spectrometry. According to separation techniques, mass spectrometry can be subdivided into gas chromatography-mass spectrometry, liquid chromatography-mass spectrometry (LC-MS), capillary electrophoresis-mass spectrometry, Fourier transform-mass spectrometry, and ultra-performance liquid chromatography. These platforms produce spectra or chromatograms that consist of thousands of peaks, each corresponding to one or more unique compounds (for mass spectrometry) or part of a single compound (for NMR). The platforms have their own reference databases containing mass spectrometry or NMR spectra of pure compounds; they are used for spectral deconvolution to determine the spectral peaks that are matched to specific chemical compounds (34–36). Several statistical methods and pathway analyses have been developed to determine compounds or spectral peaks that have changed significantly (sample-wise or group-wise) (37–39). The readers are referred to these two previous reviews (34, 40) on high-performance data processing tools. Bulk metabolomics has been successfully applied to early cancer detection, cancer monitoring and therapy screening, and cancer drug resistance (41–44). Despite these successes, an overall profile *via* bulk metabolomics (**Figure 2**, left panel) may obscure the true signals involved in the tumorigenesis or therapeutic resistance of a rare cell population; it is crucial to note that the metabolic programs are highly heterogeneous among tumor cells.

### 2.2 Single-cell metabolomics

Unlike single-cell RNA-seq, which has been widely used for many years, single-cell mass spectrometry techniques are in their infancy stages with the limited applications. To date, the reported single-cell mass spectrometry techniques include, but are not limited to: secondary ion mass spectrometry (SIMS) (45), matrix-assisted laser desorption/ionisation mass spectrometry (MALDI-MS) (46), laser ablation electrospray ionisation mass spectrometry (47), live-single cell video mass spectrometry (48), single-probe mass spectrometry (49), and T-probe mass spectrometry (50). The precision of single-cell mass spectrometry data relies heavily on how to isolate individual target cells from a solid tissue or how to pick up individual cells from a cell suspension. The commonly used single-cell isolation methods include laser microdissection, microfluidics, fluorescence-based cell sorting, limiting dilutions, and manual cell-picking with a micromanipulator (**Figure 2**, middle panel); the choice of these methods largely depends on the source of the target cells. Depending on the downstream metabolomics analytical platform, the isolated single cells can be either sampled directly for metabolomics analysis or subjected to culturing prior to downstream analysis. Single-cell mass spectrometry has been combined with machine learning models to predict the drug-resistant cancer cell phenotype (51). Other applications of this technology include: the detection of the occurrence of significant cell-to-cell differences within the neuronal cell (52, 53), identification of metabolic differences between cancer stem cells (CSCs) and non-CSCs at a single-cell resolution (54), and discrimination between breast cancer subtypes (55).

### 2.3 Spatial metabolomics

Spatial metabolomics has recently attracted increased attention as a novel technique for exploring the molecular interactions and histological heterogeneity. It enables the quantitative, qualitative, and localisation analysis of metabolites from three dimensions *in situ* within a sample from different biological tissues and organs, using MSI techniques, thus optimising and complementing traditional metabolomics approaches. The histology of cancer tissues is highly dynamic and complex (56, 57). Spatial metabolomics is unique in preserving the spatial information of metabolites by simultaneously measuring a large number of small molecules *in situ*. Most spatially-resolved metabolomics studies employ ionisation techniques coupled with MSI to create images of metabolite distribution. The main instruments used for spatial metabolomics are summarised in **Table 1**.

**Table 1:** Comparison of technologies used for spatial metabolomics.

Techniques (ionisation methods)	MSI types	Spatial resolution	Advantages	Limitations
Air flow-assisted desorption electrospray ionisation (AFADESI)-MSI (28)	ambient	~100 μm	ambient operating conditions; minimum samples preparation; improved sensitivity and spatial resolution from DESI	low reproducibility of results due to complex parameters
Atmospheric pressure matrix-assisted laser desorption ionisation (AP MALDI)-MSI (58)	vacuum	~1.4 μm	high spatial resolution; molecular information from 3D surfaces	low coverage and low sensitivity
Desorption electrospray ionisation (DESI)-MSI (59)	ambient	~50-200 μm	high-throughput; ambient operating conditions; minimum sample preparation; quick results	low spatial resolution and sensitivity
Matrix-assisted laser desorption ionisation (MALDI)-MSI (60)	vacuum	10 μm	high spatial resolution and mass resolution; suitable for examining small samples; reliable results	extra preparation steps and vacuum condition
Nanospray desorption electrospray ionisation (nano-DESI)-MSI (61)	ambient	~10 μm	ambient operating conditions; improved sensitivity and high spatial resolution	instability of the nano-DESI probes and alignment difficulty
Secondary ion mass spectrometry (SIMS)-MSI (62)	vacuum	50 nm ~ 200 μm	subcellular imaging; simple sample preparation procedure	capabilities of biochemical imaging to be improved

The spatial metabolomics techniques mainly originate from either matrix-assisted laser desorption/ionisation-MSI (MALDI-MSI) or desorption electrospray ionisation-MSI (DESI-MSI). The imaging process involves the virtual separation of the sample into many “pixels”, each described by a mass-to-charge (m/z) spectrum (63). Specialised analysis tools are used to form clusters of labelled pixels with similar metabolite signals (64–66) and generate one image per sample (**Figure 2**, right panel). In short, the metabolites are detected by pixels in order to preserve the spatial information. Notably, MALDI and DESI use different ionisation principles, each with unique advantages (**Table 1**).

In parallel with advances in spatial metabolomics techniques, recent years have seen an active development of computational methods. The typical data analysis workflow, which includes signal preprocessing, statistical analysis, visualisation, and molecular identification, validation and interpretation is similar to that used for bulk metabolomics, with an increased focus on the last three analyses in recent years. A classification scheme, using high mass resolution MALDI-MSI combined with K-means clustering analysis, has been proposed to stratify patients with gastric cancer (29). A computational multimodal spatial correlation image analysis workflow has been developed for immunohistochemistry-guided *in situ* metabolomics on intact tissue sections, thus allowing for comprehensive analyses of metabolic heterogeneity (67). A three-dimensional spatially-resolved metabolomic profiling framework has been introduced to map out the spatial organisation of metabolic fragments and protein signatures in immune cells from human tonsils (68). Spatial metabolomics is also used to assess neoadjuvant therapy in NSCLC patients (30).

### 2.4 Spatial single-cell metabolomics

Motivated by a combination of single-cell metabolomics and spatial metabolomics, the use of spatial single-cell metabolomics starts to gain popularity (69). Owing to its increased sensitivity and accuracy, MSI plays a key role in advancing spatial single-cell metabolomics detecting and quantifying metabolites with high spatial resolution profiles *in situ*. Advances in single-cell metabolomics have allowed the evaluation of spatially-resolved mass spectrometry images at the single-cell level. Various spatial single-cell metabolomics techniques have been proposed, indicating the superiority of getting enough metabolic profiles to unveil cell types, locations and associated molecular changes with different conditions (70). However, computational approaches for spatial single-cell metabolomics face a multitude of challenges, such as dealing with the batch effects that minimise confounding factors, deconvolution of high-dimension spatial resolution data, extraction of hidden molecular features (and cell subpopulations as well) from the signal noises, and linking of cell types to the cell metabolic states of the tissues.

### 2.5 Data-mining tools for mass spectrometry imaging

We enumerate open-source MSI tools (**Table 2**), with two aims. The first aim is to make it easier for biologists to navigate tools available for MSI data mining. The second aim is to give inspirations to methodology developers. *METASPACE* is a web-based application used to identify, visualise, and analyse metabolites and lipids; additionally, it consists of a public molecular annotation knowledgebase intended for spatial metabolomes (76). It takes as inputs user-submitted data in the centroided format (i.e., imzML), for which online browsing and sharing of ion images are supported for annotated metabolites and lipids, followed by signal preprocessing, data analysis, visualization, and molecular identification. As a stand-alone software*, MSiReader* provides a rich graphical user interface for data visualisation, signal processing, and unsupervised analysis of imaging mass spectrometry data (79). It supports the imzML format and can be used on any operating system (if the Matlab environment is also supported). *Cardinal* is an R package that implements statistical analysis methods, such as spatial segmentation, classification, and class comparison (71). It requires basic knowledge of R because no graphical interface is provided. *SEAM* is a platform that combines experiments and computational algorithms to quantitatively characterise metabolic intra- or inter-cellular features (84). It relies on SIMS to provide a multiscale spatial resolution, including single-nucleus segmentation, single-nucleus representation, and differential metabolite analysis. *SpaceM* is a newly developed method for spatial single-cell metabolomics to unveil the relationship between metabolism and phenotype at the single-cell level; it can examine the native spatial context of metabolites and characterise metabolic heterogeneity at the single-cell level (85). The readout comprises the metabolic profiles, fluorescence intensities, and spatio-morphological features.

**Table 2:** The imaging-based mass spectrometry tools.

Software	Categories	Techniques (platforms)	Implementation	Availability
BioMap	Platform	MALDI-MSI	IDL™	http://www.maldi-msi.org
Cardinal (71)	Statistics	MALDI-MSI/DESI-MSI	R	https://www.cardinalmsi.org
ColocML (72)	Statistics	MSI	Python	https://github.com/metaspace2020/coloc
Datacube Explorer (73)	Platform	MSI	C# (.NET)	https://amolf.nl/download/datacubeexplorer/
MassImager (74)	Platform	AFADESI-MSI	C++	http://www.chemmind.com/en/support_download.html
massPix (75)	Annotation	MSI	R	https://github.com/hallz/massPix
METASPACE (76)	Platform	MALDI-MSI/DESI-MSI	Web-application	http://metaspace2020.eu/
microMS (77)	Platform	microscopy-guided MSI	Python	https://neuroproteomics.scs.illinois.edu/microMS.htm
MIRION/Imaging3D (78)	Statistics	AP-SMALDI10 MSI	MATLAB	https://www.nature.com/articles/nmeth.4433#MOESM5
msIQuant (77)	Statistics	MALDI-MSI	C++	http://www.maldi-msi.org
MSiReader (79)	Statistics	MSI	MATLAB	https://www.msireader.com/
OpenMSI (80)	Platform	MSI	Web-application	http://openmsi.nersc.gov
OpenMZxy (81)	Platform	LTP-MSI	Python	https://bitbucket.org/lababi/openmzxy/src/master/
pySM (82)	Annotation	MALDI-MSI/SIMS	Python 2.7	https://github.com/alexandrovteam/pySM
rMSI (83)	Platform	MSI	R	https://github.com/prafols/rMSI
SEAM (84)	Platform	SIMS	Python + MATLAB	https://doi.org/10.5281/zenodo.5025068
SpaceM (85)	Platform	MALD-MSI	Python	https://github.com/alexandrovteam/SpaceM
SPUTNIK (86)	Statistics	MSI	R	https://github.com/paoloinglese/SPUTNIK

## 3 Improved understanding of the metabolic mechanisms underlying cancer drug resistance

**Figure 3:**
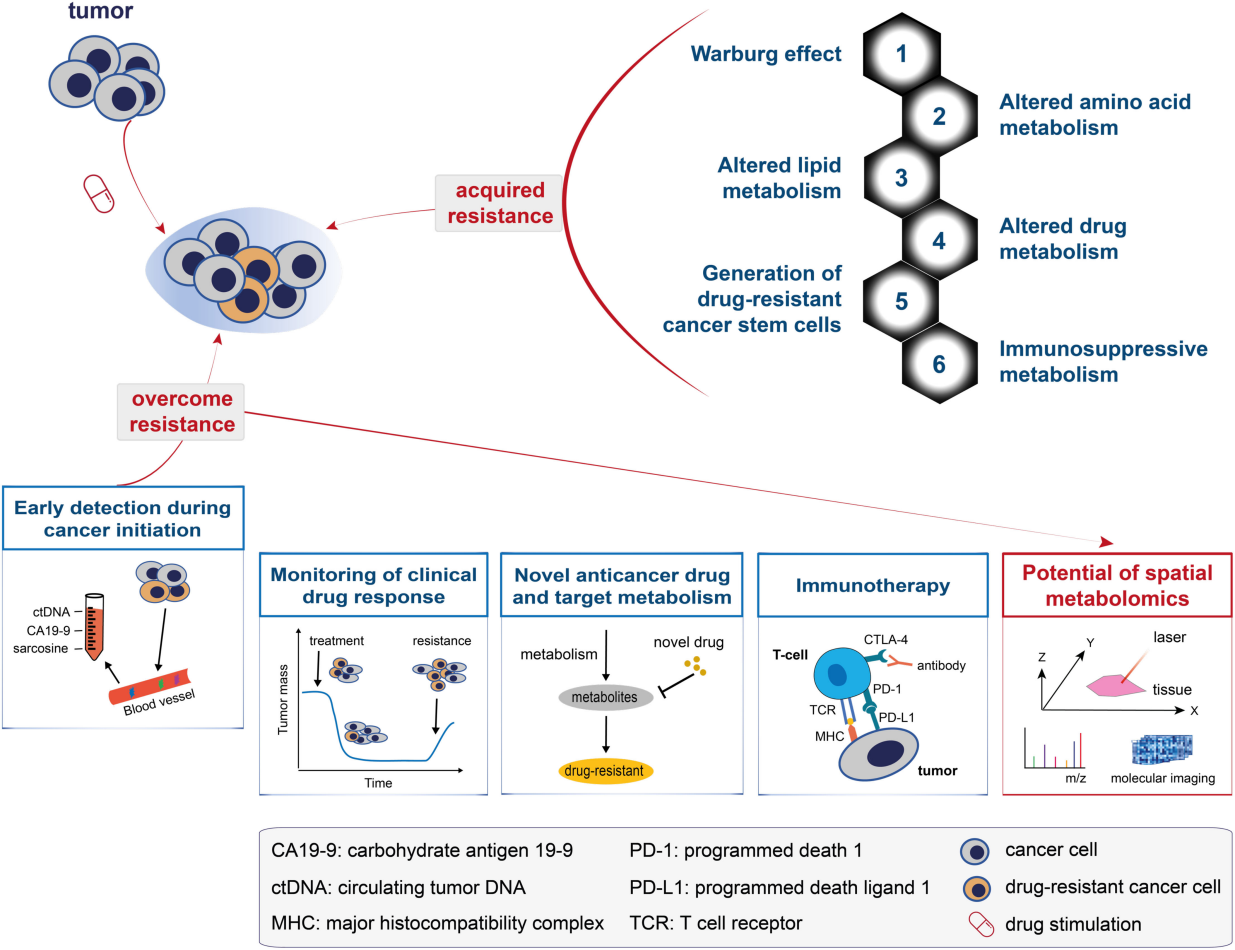
A model for understanding and overcoming cancer drug resistance. The top panel shows the metabolic changes that drive cancer drug resistance and can be generalised into: the Warburg effect, altered amino acid metabolism, altered lipid metabolism, altered drug metabolism, generation of drug-resistant cancer stem cells, and immunosuppressive metabolism. The bottom panel shows the five solutions proposed to overcome cancer drug resistance, namely: early detection during cancer initiation, monitoring of clinical drug response, novel anticancer drug and target metabolism, immunotherapy, and the potential of spatial metabolomics in overcoming cancer drug resistance.

**Figure 4:**
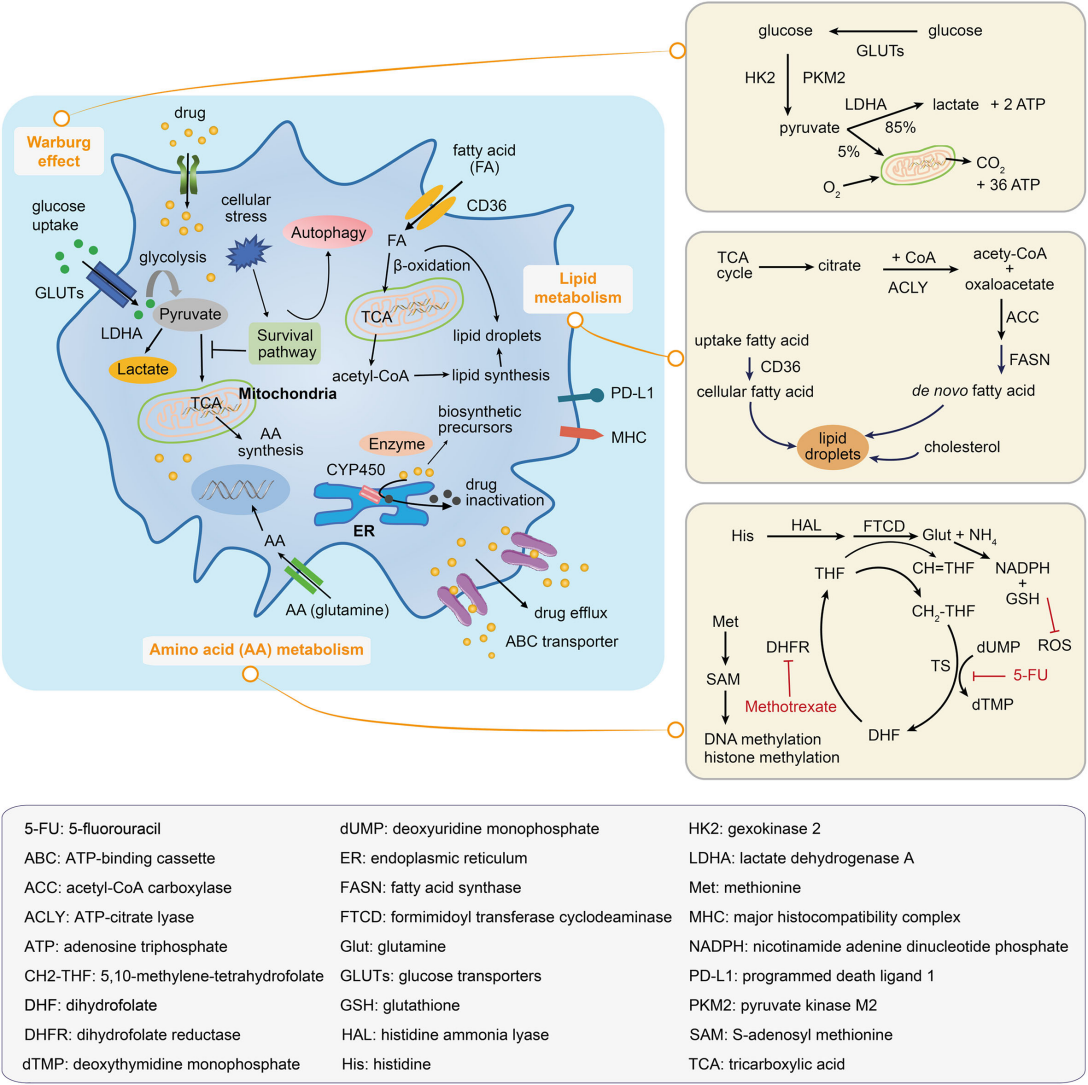
Metabolic mechanisms illustrated within a drug-resistant cancer cell. The drug-resistant cancer cell increases the glucose uptake and represses the tricarboxylic acid (TCA) activation in order to satisfy the energy needs; this leads to the accumulation of lactate and an increase in the fatty acid absorption, which can be oxidated to generate biosynthetic precursors. Amino acid metabolic reprogramming maintains tumor progression and supports the survival of drug-resistant cancer cells. Inside of the drug-resistant cell, the drug may be inactivated by decomposition to form biosynthetic precursors or by its transportation to the extracellular environment. The right panels illustrate the Warburg effect, the lipid droplets and fatty acid synthesis, and the amino acid metabolic pathway.

Drug resistance is a major cause of cancer therapy failure. Despite considerable efforts over the past decades, knowledge about the mechanisms involved in drug resistance remains largely unknown. Metabolomics has offered novel insights into these mechanisms in cancer patients (87, 88). The metabolic alterations and mechanisms associated with drug resistance have drawn increasing attention and can be generalised into the following aspects (top-right panel in **Figure 3**; also see **Figure 4**): the Warburg effect, altered amino acid metabolism, altered lipid metabolism, altered drug metabolism, generation of drug-resistant cancer stem cells, and immunosuppressive metabolism.

### 3.1 The Warburg effect

In addition to promoting tumorigenesis, metabolic alterations in cancer cells provide an environment that tends to increase drug resistance. Glucose transporters, glycolytic enzymes, and stress responses are potential mechanisms by which the glycolytic pathway (Warburg effect) can confer a chemo-resistant phenotype.

The entry of glucose into the cells is facilitated by a transporter family known as the glutamines (GLUTs) (89), with three members (*GLUT1/3/4*) widely studied in cancer. A study using lung and breast cancer models revealed a potent anti-tumor effect of WZB117, a *GLUT1* inhibitor, that could decrease glycolysis and reduce intracellular adenosine triphosphate (ATP) levels (90). In another study, an anti-viral drug ritonavir, known to inhibit *GLUT4* (91), inhibited the proliferation of primary multiple myeloma cells (92). Drug resistance acquired in glioblastoma cells was reported to be associated with an increase in the major neuronal glucose transporter *GLUT3* (93).

Glycolytic enzymes have also been implicated in promoting a drug-resistant phenotype. Two genes, *HK2* and *PKM2*, encode the first and final rate-limiting enzymes in the glycolytic pathway, respectively. Both genes are known to be up-regulated in cancer to induce drug resistance (94). The gene *LDHA* encodes an enzyme that converts pyruvate into lactate (the end-product of glycolysis; **Figure 4**), and increased levels have been shown to confer resistance to trastuzumab in breast cancer (95).

Nutritional deficiency promotes cellular adaptation to stress by activating survival signals and leading to drug-resistant phenotypes. Glucose deprivation can activate the heat shock factor (HSF1) to regulate the heat shock response (96). HSF1 can increase glucose uptake and promote glycolysis and cellular adaptation to stress. A higher level of HSF1 has been reported in trastuzumab-resistant cells, whereas inhibition of HSF1 led to the sensitisation of the cells to trastuzumab (97). Furthermore, autophagy is associated with glucose deprivation. It is believed that autophagy can promote cell survival by recycling intracellular organelles to produce energy; autophagic cells are highly resistant to drug treatments (**Figure 4**) (98).

### 3.2 Altered amino acid metabolism

The role of amino acids has been gaining popularity in the field of cancer metabolism in recent years. Cancer cells reprogram the amino acid metabolism to maintain tumor progression and support the complex microenvironment in driving the resistance to anticancer therapies. Amino acid provides resistant cells with drug-specific adaptations, drive the epigenetic modulation of drug-resistant cancer cells, modulate the tumor microenvironment to anticancer therapies, mediate tumor immune evasion, and regulate CSCs to promote cancer aggressiveness.

Amino acid metabolism provides resistant cancer cells with specific adaptive mechanisms to counteract anticancer drugs (**Figure 4**). Cancer cells generally display metabolic adaptations during tumor genotoxic therapies by up-regulating nucleotide biosynthesis to prevent DNA damage-induced cell death (99). For instance, in cisplatin-resistant NSCLC cells, glutamine is mainly used for nucleotide biosynthesis (100). In methotrexate-resistant hematopoietic malignant cell types (Burkitt’s lymphoma cells, and chronic myeloid leukemia cells), depletion of formimidoyltransferase cyclodeaminase and histidine ammonia lyase favored therapy resistance by decreasing the histidine catabolism that consumed the cellular pool of tetrahydrofolate and by increasing the nucleotide synthesis under methotrexate treatment (101). In addition, dietary methionine restriction was reported to produce therapeutic responses in patient-derived xenograft models of 5-fluorouracil-resistant RAS-driven colorectal cancer. Under dietary methionine restriction, cancer cells increased the production of methionine from homocysteine to consume the intracellular 5,10-methylene-tetrahydrofolate, thereby affecting the folate cycle-related metabolites and nucleotide biosynthesis (102). Several anticancer drugs rely on increased oxidative stress to mediate cell death. Alternatively, the resistant cancer cells adapt amino acid metabolism to generate the critical metabolites, such as nicotinamide adenine dinucleotide phosphate (NADPH) and reduced glutathione (GSH), to balance cellular redox homeostasis and overcome reactive oxygen species (ROS)-induced cell death. Glutamine-mediated NADPH production was found to be pivotal for maintaining the cellular redox balance in gemcitabine-resistant pancreatic cancer cells (103). Similarly, increased glutamine utilisation supported the survival of sorafenib-resistant hepatocellular carcinoma (HCC) cells by increasing the NADPH and GSH levels (104). In another study, aspartate provided the metabolic precursors for NADPH generation and decreased the induction of mitochondrial ROS in response to gemcitabine-induced apoptosis (105).

Amino acid metabolism drives the alteration of epigenetics, which supports the survival of drug-resistant cancer cells (**Figure 4**). Cellular DNA or histone-methylated modification is determined by the activities of methyltransferases and demethylases. S-adenosylmethionine (SAM; derived from methionine) is the dominant methyl donor for these enzymes. A decrease in methionine metabolism and low levels of SAM and S-adenosylhomocysteine resulting in DNA hypomethylation with overall genome were observed in paclitaxel- or taxane-resistant triple-negative breast cancer (TNBC) cells (106). Furthermore, a significant increase in alpha-ketoglutarate, a tumor metabolite, supported the activities of histone lysine demethylases and favored the emergence of dedifferentiated BRAF inhibitor-resistant subpopulations (107).

Amino acids play an important part in the complex crosstalk of the tumor microenvironment and the regulation of tumor-induced immunosuppression, thus leading to cancer drug resistance. Cancer cells have a specific mechanism to educate the neighboring stromal cells to adapt their metabolism. In pancreatic ductal adenocarcinoma, cancer cells were reported to promote pyrimidine synthesis by the tumor-associated macrophages (TAMs), leading to an increase in the production of deoxycytidine by macrophages, which directly competes with gemcitabine and hinders its efficiency as a treatment drug for cancer cells (108). In addition, the melanoma was reported to weaken immunotherapy by releasing Wnt5a, which induced indoleamine 2,3-dioxygenase 1 activity in dendritic cells and reduced the efficacy of programmed cell death protein 1 (PD-1) blockade therapy (109). Furthermore, glutamine metabolism in cancer cells affects the recruitment of myeloid-derived suppressor cells (MDSCs, an immunosuppressive environmental factor) and decreases inflammatory TAMs, leading to checkpoint blockade-resistant tumors to be immunosuppressive (110).

CSCs pose a major challenge in cancer therapy owing to their intrinsic characteristic of decreased response to drug treatment. Amino acid metabolism promotes the escape of CSCs from drug treatment. For example, amino acid metabolism fuels oxidative phosphorylation (OXPHOS) to prevent chemotherapy toxicity in leukemia stem cells (111). Early studies revealed that methionine metabolism regulates the maintenance and differentiation of human pluripotent stem cells (112). Similarly, methionine metabolism may enhance the stemness of CSCs, which could result in poor outcomes following clinical treatment. Methionine restriction may reduce the CD44^high^/CD24^low^ CSCs population in TNBC cell lines, leading to the inhibition of methionine adenosyltransferase 2A, which is responsible for SAM biosynthesis in CSCs (113). Likewise, methionine is a metabolic dependency of tumor-initiating cells (also called CSCs), which are derived from resected primary NSCLCs that exhibit increased methionine cycle metabolites, thus becoming addicted to exogenous methionine (114). These findings indicate that amino acid metabolism can shape the tumor environment of CSCs and lead to the aggressiveness for drug-resistant cancer cells.

### 3.3 Altered lipid metabolism

Lipid metabolic alterations frequently occur in malignant tumors, and thus, understanding the mechanisms that maintain lipid homeostasis in drug-resistant cancer cells may reveal the metabolic characteristics that could be applied in the clinical setting. Cancer-associated lipid metabolic alterations, which includes increased lipogenesis, lipid catabolism and enhanced lipid storage from lipid droplets, can drive tumor resistance to drug treatment (**Figure 4**). *De novo* lipid biosynthesis drives tumor resistance. Compared to normal cells, cancer cells tend to increase lipogenesis rather than depending on dietary lipids or using lipids synthesised from liver cells. Aberrant *de novo* fatty acid biosynthesis provides a continuous supply of resources for membrane synthesis, energy consumption, and signaling to cancer cells. Fatty acid biosynthesis depends on a series of enzymes, including but not limited to: ATP-citrate lyase (ACLY), Acetyl-CoA carboxylase (ACC), and fatty acid synthase (FASN). ACLY was reported to be upregulated in BRAF-mutation melanoma and promoted mitochondrial biogenesis and OXPHOS to support tumor growth and resistance to vemurafenib (115). ACC is the rate-limiting enzyme of the fatty acid biosynthetic process; dysfunction of ACC in head and neck squamous cell carcinoma allowed cancer cells to convert glycolysis to lipogenesis, thus resulting in resistance to cetuximab (116). Increased expression and activity of FASN, another rate-limiting enzyme in lipogenesis, were reported to increase the tolerance of cancer cells to chemotherapeutic drugs, such as gemcitabine in pancreatic cancer (117) and cisplatin in ovarian cancer (118). Moreover, high levels of FASN were found to be inversely correlated with survival prognosis (119).

Fatty acid uptake and consumption promote therapy resistance. Fatty acid can enter the cancer cell *via* lipid transporters and fuel its oxidation in the mitochondrial to produce energy for cell survival. In HER2+ breast cancer cells, lapatinib-resistant cells increased the expression of the fatty acid transporter, CD36, to absorb abundant exogenous fatty acid and lipids, reshaping the metabolic programs that allow cancer cell survive during nutrient starvation (120). In ovarian cancer, resistant cells could be resensitised to cisplatin by lipid deprivation, thus exhibiting the role of exogenous lipids and cholesterol uptake in drug-resistance (121).

Lipid droplets play an essential role in cancer cells and are increased when sensing cellular stress induced by anticancer treatment (**Figure 4**), associated with tumor aggressiveness and therapy resistance. A recent study demonstrated that hypoxia drives lipid droplet formation *via* exogenous lipid droplet uptake and facilitates its accumulation in clear cell renal carcinoma and colorectal cancer cells (122). HIF1 was reported to regulate the expression of AGPAT2 (encoding an enzyme involved in the triacylglycerol biosynthetic pathway) in HCC and cervical adenocarcinoma cells. AGPAT2 induction was necessary for hypoxia-dependent lipid droplet accumulation, which correlated with etoposide resistance. Furthermore, AGPAT2 knockdown reduced the lipid droplet content and reverted the resistance to therapy in cervical adenocarcinoma cells (123). Additionally, lipid droplets are known to accumulate in breast cancer cells that are resistant to tamoxifen, although the mechanism involved in this accumulation is largely unknown.

### 3.4 Altered drug metabolism

Drug-resistant cancer cells acquire a set of unique metabolic mechanisms to fight against chemotherapeutic agents. Enzymes are the major factors that determine the concentration of therapeutic drugs inside and outside cells. Enzymes utilise drug metabolism to resist drug treatment *via* two methods: one is drug degradation of enzymes (such as *via* oxidation, reduction, and hydrolysis) to reduce the activation of prodrugs; the other is the conversion of the drugs into intermediate metabolites for macromolecule synthesis (**Figure 4**). Detoxification by cytochrome P450 is an example of drug degradation by enzymes; drug resistance with increased activity of cytochrome P450 resulting in the docetaxel inactivation has been reported in breast cancer (124). In another study, increased glutathione production mediated by glutathione transferases was found to be critical for the resistance of cancer cells to platinum-based anticancer drugs, such as cisplatin (125).

### 3.5 Generation of drug-resistant cancer stem cells

Chemotherapeutic agents can impair a large number of tumor cells. These agents can be transported or removed from drug-resistant CSCs *via* various mechanisms following drug stimulation or selective pressure. For instance, the ATP-binding cassette (ABC) and drug transporters (such as P-glycoprotein) have been reported to be overexpressed in mitoxantrone-resistant cells; they act to remove chemotherapeutic agents (126). Extruding a variety of compounds from cells, the ABC transporter presents an obstacle in treating chemotherapy-resistant cancers (**Figure 4**). CSCs share the following characteristics associated with normal stem cells: being quiescent for a long time and self-renewing, resistance to drugs through the up-regulation of drug efflux transporters, low metabolic activity, and resistance to apoptosis with enhanced activity of DNA repair enzymes (126, 127). Thus, CSCs can remain stable during patient recovery owing to these stemness-like characteristics. Alternatively, they can metastasise to distant organs, leading to cancer recurrence. Therefore, it is of great value to identify and eliminate these small populations of cancer cells to overcome drug resistance. Metabolomics, especially spatial metabolomics, might aid in detecting the precise location of CSCs.

### 3.6 Immunosuppressive metabolism

Immune checkpoint therapy using, for example, two FDA-approved drugs (nivolumab and pembrolizumab), represents new forms of cancer therapy. Immune functions are related to tumor cell metabolism; for example, gain-of-function mutations in isocitrate dehydrogenase (IDH) resulted in the production of D-2-hydroxyglutarate, an oncometabolite that altered T-cell metabolism to impair CD8+ T cytotoxicity and interferon-gamma signaling in patients with IDH1 mutant gliomas (128). Down-regulating the cellular metabolism can weaken the ability of an immune system to inhibit tumor growth. In other words, altering the metabolism might contribute to immune resistance in drug-resistant tumors. In the tumor microenvironment, immune cells are at a metabolic disadvantage owing to the limited availability of carbon nutrients due to competition from the tumor cells (129). Cisplatin-resistant cancer can undergo a “second” metabolic switch that favors oxidative metabolism to increase amino acid uptake (130). Consequently, the cytotoxic effector T-cells are deprived of amino acids; being highly anabolic requires a large number of amino acids needed for growth (131, 132). For instance, kynurenine, a product of tryptophan catabolism, inhibits T cell activation (a cytolytic function) and supports the differentiation of immunosuppressive regulatory T-cell (131). Hypoxia-induced HIF1α can promote the expression of programmed cell death-ligand 1 (PD-L1) in MDSCs and mediate effective immunosuppressive activities in cancer-specific effector T-cells (133). Similarly, the depletion of arginine by arginase in MDSCs was found to be involved in immune resistance (134). The metabolism of lipids and fatty acids was reported to be involved in the immunosuppressive tumor microenvironment (135). PD-1 and PD-L1 are widely used in anti-tumor immune therapy. PD-1 is mainly expressed in activated T-cells, whereas PD-L1 is often seen in cancer cells. The engagement of both PD-1 and PD-L1 suppresses the function of effector T-cells. Consistent with early reports that cisplatin treatment can induce PD-L1 expression in NSCLC, overexpression of PD-L1 has been associated with poor outcomes in cancer patients (136). However, little is known about the specific mechanisms involved in this process. Cisplatin-resistant NSCLC can undergo an epithelial-mesenchymal transition to enable invasion/metastasis and escape immune checking by maintaining a higher level of PD-L1 (136). Therefore, monotherapy with immune checkpoint inhibition is thought to be insufficient for the treatment of drug-resistant cancers. It is necessary to identify and precisely locate tumor cells and immune cells in the tumor microenvironment using metabolomics technologies, especially spatial metabolomics. In summary, alterations in metabolic products in drug-resistant cancers can reshape the tumor microenvironment and modulate immune function, thus representing an ideal direction for developing therapeutics.

## 4 Progress and potential of metabolomics in overcoming cancer drug resistance

Changes in the metabolome are most closely related to the drug-resistant phenotype (17). Five metabolic solutions to overcome cancer drug resistance are discussed in the following subsections (**Figure 3**, bottom panel): early detection during cancer initiation, monitoring of clinical drug response, novel anticancer drug and target metabolism, immunotherapy, and the potential of spatial metabolomics in overcoming cancer drug resistance.

### 4.1 Early detection during cancer initiation

Chemoresistant cancers tend to display an aggressive clinical outcome and early recurrence. During drug treatment, a small subpopulation of cancer cells can remove the anticancer drug, and thus the developing resistant cells become the dominant population. Screening the subset of chemoresistant cancer cells at an early stage might prove effective in inhibiting cancer drug resistance. Metabolomics is a powerful tool that can unbiasedly identify cancer biomarkers that drive cancer drug resistance.

Metabolites are stable in the serum; hence, it is possible to establish a noninvasive method for the early detection of the metabolic biomarkers of cancer. Conventional methods for the detection of pancreatic cancer include the estimation of the serum level of the carbohydrate antigen 19-9 (137). The potential role of sarcosine in prostate cancer progression has been revealed by profiling metabolites from a large cohort of clinical specimens (138). Total choline levels are consistently up-regulated in breast cancer and can be used to differentiate between cancer and normal tissues (139). Prostate-specific antigen or the prostatic fluid levels can be used for the early detection of prostatic cancer (140). The first urine metabolomics screening test for colon cancer, called Polyp, which uses a defined diagnostic metabolomic profile to identify colonic adenomas, was recently released (141).

Circulating tumor DNA (ctDNA) tests can be used for early detection, including non-invasive dynamic detection of cancer and monitoring of clonal evolution (142). It can be utilised for selecting the subsets of cancer patients, especially the high-risk populations. The ctDNA and metabolites detection can be coupled with a flexible therapeutic method to prevent relapse. However, clinical screening studies based on larger cohorts are required to confirm the efficacy of metabolites and ctDNA screening.

### 4.2 Monitoring of clinical drug response

Routine approaches to assess drug resistance during chemotherapy treatment have been used; for example, seven metabolites (hypotaurine, uridine, dodecanoylcarnitine, choline dimethylglycine, niacinamide, and L-palmitoylcarnitine) have been identified to be associated with chemoresponses (143). In one study, the metabolic profiles of patient-derived tumor xenografts revealed metabolic biomarkers that were associated with the resistance to five anticancer drugs (41).

New technologies allow for the monitoring of the extent of drug resistance at an early chemotherapeutic stage from a spatial or single-cell dimension in their native microenvironment. An analytical approach that combines single-cell metabolomics with machine learning models has been developed to address chemotherapy-induced drug resistance challenges (50). These models can rapidly and accurately predict the different degrees of drug resistance within a single live cell, which can be potentially employed to assess chemotherapeutic efficacy in the clinic (50). Spatial metabolomics technologies can be used to monitor the drug resistance appearance *in situ*. Resected NSCLC tissue specimens obtained after neoadjuvant chemotherapy were subjected to high-resolution mass spectrometry, and the data generated was used to develop an approach for evaluating the response to neoadjuvant chemotherapy in patients with NSCLC (30). Specific lipid and metabolic profiles of R-CHOP-resistant DLBCL have been generated to obtain information about the analyte composition and molecular distributions of therapy-resistant and sensitive areas. The spatial metabolomics techniques helped monitor metabolic changes by identifying the decrease in ATP and the increase in adenosine monophosphate in the R-CHOP-resistant DLBCL (27).

### 4.3 Novel anticancer drug and target metabolism

Chemotherapy remains a major approach to cancer treatment. Traditional chemotherapeutic drugs used in cancer treatment include, but are not limited to: (i) alkylating agents that target the DNA, causing either single-strand breaks or crosslinking of DNA to prevent the cell from proliferating; (ii) antimetabolites that disrupt DNA synthesis and cell division by inhibiting the formation of normal nucleotides or by direct interaction with DNA, thereby preventing the extension of DNA strands; and (iii) enzyme inhibitors that affect DNA replication and the cell cycle. As a folic analog, the antimetabolite (5-fluorouracil) is a chemotherapy drug that inhibits thymidylate synthase and decreases the thymidine triphosphate level for DNA replication. These traditional chemotherapy drugs are non-specific to tumors and may inhibit the proliferation of normal cells, causing severe side-effects, such as drug resistance. Targeting cancer metabolism pathways involved in tumor development and metastasis is increasingly becoming feasible for cancer therapy. Metabolic reprogramming is one of the hallmarks of cancer cells; altered metabolic pathways (such as glycolysis, amino acids metabolism, fatty acid synthesis, and glutamine metabolism) support the rapid proliferation of cells. Furthermore, these dysregulated metabolic features are also linked to therapeutic resistance in cancers (144).

Metabolites in cancer are linked to the activation of proto-oncogenes and the inactivation of tumor suppressor genes. Upstream regulators of metabolic pathways (such as HIF, PI3K, AKT, mTOR, and AMPK) are important targets for anticancer drugs. Targeting HIF prevents the metabolic shift or adaptation of tumor cells to hypoxia. Anticancer agents (such as PX-478) that reduce the HIF-α level have demonstrated potent anti-tumor effects (144). Rapamycin, an inhibitor of mTORC1, was reported to enhance the anti-tumor effect of cisplatin in alpha-fetoprotein-induced gastric cancer (145).

Targeting glucose metabolism is considered as one of the most important anticancer strategies for energetic limitation, as highlighted by targeting enzymes that are involved in the transport and breakdown of glucose. Inhibitors of glucose transporters (such as Phloretin and Ritonavir) have demonstrated anticancer effects by reducing the uptake of glucose and slowing down glycolysis rate (145). These agents, alone or in combination with chemotherapy, have demonstrated *in vitro* activities against cancers (such as colon cancer, leukemia, lung cancer, breast cancer, and multiple myeloma). Pyruvate kinase catalyses the conversion of phosphoenolpyruvate and adenosine diphosphate into pyruvate and ATP, respectively; it was reported to be associated with tumor growth and cisplatin resistance (145).

### 4.4 Immunotherapy

Learned from conventional chemotherapy, efforts have been shifted towards actionable strategies to combat therapeutic resistance to immunotherapy, through converting tumors from being immunologically “cold” into “hot”. This can be achieved by enhancing endogenous T-cell function (146), expanding tumor-infiltrating lymphocytes *ex vivo* (147), or administrating antigen-specific engineered T-cells (148, 149).

Combination strategies are favored to overcome drug resistance (150). A typical method to enhance the efficacy using combination therapy involves blocking the antibodies against two key immune checkpoints, cytotoxic T lymphocyte-associated antigen-4 (CTLA-4) and PD-1, thereby resulting in higher response rates to the treatment and improvements in the survival of patients with metastatic melanoma (151). Blocking the CTLA-4 may facilitate the conversion of the tumor microenvironment from being “cold” into “hot” (152). Each checkpoint inhibitor exerts both overlapping and unique effects on tumor-specific T-cells (146). Numerous strategies that combine immune modulators of the tumor microenvironment and immune checkpoint inhibitors are currently under clinical trials (153).

Molecular-targeted therapy can be used either in conjunction with immunotherapy or to help alter the tumor microenvironment in order to mimic the therapeutic effect of immunotherapy. The most illustrative is the melanoma with oncogenic BRAF. BRAF-targeted therapy alone provides limited durable disease control (154) but creates favorable effects in the tumor microenvironment; these effects include increased antigen and HLA expression, increased T-cell infiltrate, reduced immunosuppressive cytokines, and improved T-cell function (155). Therefore, molecular-targeted therapy may aid in converting the microenvironment from “cold” into “hot”, likely *via* a phenomenon called “adaptive resistance” (155). Based on insights into the T-cell and overall immune function, the strategies that enhance the response to immunotherapy include metabolic reprogramming of T-cells (156, 157) and the modulation of gut microbiome metabolites (158, 159).

### 4.5 Potential of spatial metabolomics in overcoming cancer drug resistance

Over the last decade, MSI has been increasingly applied to investigate the spatial distribution of biomolecules in tissue sections. The potential for the clinicopathologic analysis of cancer can reveal the distribution of hundreds of molecules in a single measurement, without prior derivatisation. Alterations in the metabolic processes are one of the most outstanding characteristics of tumor tissues. The study of spatial metabolomics has helped explore the etiology, properties, subtypes, and vulnerabilities of various cancers. LC-MS has contributed significantly to understanding the key mediators of cancer metabolic pathways, such as the carnitine system. However, the lack of spatial information about metabolites prevents from further exploring the heterogeneity of the cancer tissues and discovering the alteration in the tumor microenvironment. The use of MSI techniques has offered new insights into the tumor-associated metabolic reprogramming, with the applications of spatial metabolomics in cancer research described in the next subsection.

### 4.6 Application of spatial metabolomics in cancer drug resistance

Spatial metabolomics can be carried out to overcome drug resistance. Early detection to identify the metabolic changes in the tumors during the preliminary stage *in situ* and classifying the subtype of tumors by spatial metabolomics with high confidence will help in drug selection and in avoiding the occurrence of chemoresistance. Uncovering the complexity of the tumor microenvironment may help in guiding the immunotherapy methods. Spatial metabolomics can provide an unbiased estimate of the changes in metabolites *in situ* between cancer and normal cells, and between cancer and drug-resistant cancer cells for precise medicine. In this section, we discuss the recent studies on spatial metabolomics in two types of cancers (lung cancer and DLBCL, which represent a solid tumor and a blood tumor, respectively).

#### 4.6.1 Lung cancer (NSCLC)

Lung cancer is the second most common cancer worldwide and the leading cause of cancer-related deaths worldwide (42). The majority of lung cancers are NSCLCs, which comprise many subtypes, such as adenocarcinoma (ADC) and squamous cell carcinoma (SqCC). All of these subtypes contribute to the heterogeneity of the tumor. The identification and monitoring of the diverse changes in lung cancer are essential to overcome the progression into malignancy and resistance. Recently, MALDI-MSI-based metabolomics was successfully applied to reveal differences between NSCLCs and normal lung regions based on a lipid analysis (160). Furthermore, MALDI-MSI was used to confirm differences between NSCLC subtypes (161). The classification of SqCC and ADC *via* histology-guided MALDI-MSI was performed based on the metabolites, and rare IDH-mutated NSCLC was screened by evaluating the levels of the oncometabolites (161). Data generated from resected NSCLC tissue specimens were used to evaluate the response to neoadjuvant chemotherapy in patients (30). In summary, spatial metabolic profiles collected by MALDI-MSI have been used to classify tissues within the tumor microenvironment, categorise highly similar cancer subtypes, and monitor the response to chemotherapy.

#### 4.6.2 DLBCL

DLBCL is the most common subtype of non-Hodgkin lymphoma. Although many patients are cured with standard chemoimmunotherapy, up to 40% of DLBCL patients have refractory disease or develop relapse following R-CHOP or similar regimens, warranting the development of novel, more effective therapeutic strategies for these patients (162). To understand the molecular mechanisms underlying relapsed DLBCL, Florian et al. studied differences in the lipid and metabolic compositions of nontreated and R-CHOP-resistant tumors using a combination of *in vivo* DLBCL xenograft models and MSI (27). In another study, Anthony et al. used imaging mass cytometry on 33 cases of DLBCL to characterise the tumor and immune cell architecture and correlate it to clinicopathological features, such as the cell of origin, gene mutations, and responsiveness to chemotherapy (163). Notably, the spatial metabolomic technology is performed only in the lymph nodes in DLBCL due to its relatively stable position within the tissue.

#### 4.6.3 Other cancers

We also briefly review other findings from spatial metabolomics in breast cancer, esophageal cancer, and glioblastoma. Breast cancer is one of the most commonly diagnosed cancers in women. It is one of the most highly heterogeneous cancers because it consists of many different types of malignancies originating from different cells or tissues. Hence, several groups have used MSI to visualise the multiple aspects of metabolic alterations in various types of breast cancer. Two studies reported the use of DESI-MSI to detect metabolite information and distinguish tumor tissues from normal tissues (164, 165). Sun et al. examined alterations in energy consumption using breast cancer tissues by investigating the spatial alterations in carnitines, which are the key regulators and transporters involved in fatty acid, carbohydrate, and lipid metabolisms with MALDI-MSI (166). A surge in the incidence of esophageal cancer has been observed over the past few decades, and the disease continues to have a poor prognosis (167). In a recent study, Abbassi-Ghadi et al. focused on spatially-resolved lipid analysis to identify invasive esophageal adenocarcinoma at an early stage from several premalignant tissues (117). In another study, airflow-assisted desorption electrospray ionisation-MSI (AFADESI-MSI) was used to acquire region-specific metabolites from 256 esophageal cancer tissues (28). Glioblastoma, a common type of brain tumor, is one of the most fast-growing and aggressive cancers; matrix-assisted Laser desorption/ionisation time of flight-MSI (MALDI-TOF-MSI) has been used to compare metabolites between normal tissues and different subtypes of glioblastomas (168).

## 5 Outstanding challenges and future perspectives of integrative spatial omics approaches to dissect cancer drug resistance

MSI technologies enable spatial metabolomics to measure the metabolites *in situ*, but are limited by the sensitivity of the detection, annotation, quantification, and spatial resolution. This limitation can be partially solved by combining spatial and bulk metabolomics, thus providing more comprehensive coverage of the metabolic profiles. The critical technical issue in MALDI-MSI is how to deal with image-abundant metabolites without loss of sensitivity while maintaining a high spatial resolution. Some classes of lipids are difficult to image with conventional MALDI-MSI (169). Thus, it is necessary to improve the sensitivity and accuracy using advanced computational bioinformatics systems.

Multi-omics (such as transcriptomics, proteomics, and metabolomics) can be applied to unveil the tissue complexity, heterogeneity, intracellular signaling, and disease progress underlying the drug resistance. For example, the concordance between gene expression and staining of the corresponding proteins can be assessed in the same tissue regions to validate the findings of spatial transcriptomics (170, 171). Furthermore, models based on spatial transcriptomics data alone are not a direct reflection of the metabolic activities. Hence, it is important to incorporate the information from other spatially-resolved omics techniques, such as metabolomics and proteomics (172). Combining spatial metabolomics and single-cell metabolomics might prove helpful in exploring the tumor heterogeneity and the surrounding environment (70). The identity of cells (e.g., tumor cells, immune cells, fibroblasts, and drug-resistant cells) can be characterised by single-cell metabolomics, while the location information can be resolved by spatial metabolomics. Consequently, it becomes possible to study, in greater detail, how tumor cells interact with adjacent cells or their surrounding cell environments; additionally, it enables the identification of the cells targeted by the drug treatment. However, the combination of spatial metabolomics and single-cell metabolomics is challenging, both technically and computationally. Advanced methods supporting such integration are much needed to dissect cancer drug resistance; we are on the agenda extending our previously established approaches (173–179) to do so in the near future.

## 6 Conclusion

Metabolic reprogramming has been recognised as a hallmark of cancer in promoting therapy resistance and many others. Targeting metabolic alterations in cancer cells and host patients represents an emerging therapeutic strategy for overcoming cancer drug resistance, particularly at the advent of spatial metabolomics. This review describes the latest progress in technologies and methods currently available for (bulk, single-cell and/or spatial) metabolomics, and discusses how these latest advances have improved our understanding of the metabolic mechanisms underlying tumor responses to anticancer drugs, along with the potential of using metabolomics to overcome drug resistance and the perspective on further developing integrative spatial omics approaches to dissect cancer drug resistance.

More specifically, we have discussed the specific metabolic programs and adaptations that exist in drug-resistant tumors, how these adaptations depend on both the drug and the origin of the tumor, and how they contribute to drug resistance. Accumulated evidence strongly suggests that combining many first-line chemotherapeutic agents with metabolic drugs holds great promise in increasing the drug efficacy. Moreover, a better understanding of the altered metabolism in different drug-resistant cancers and the distribution of metabolites and the tumor microenvironment through spatial metabolomics is essential to further improve the outcomes of cancer therapy. This additional information will provide insights into the molecular mechanisms of resistance, which will help in identifying novel metabolic targets that can be used for combined treatment. Finally, this knowledge may be applied to identify the prognostic biomarkers for drug response, which could drive current therapies by predicting the drug response based on the metabolic state of the tumor, thereby contributing to more effective personalised medicine.

## Author contributions

ZZ and HF: Conceptualisation. ZZ, CB and HF: Data curation and writing - original draft preparation. ZZ, CB and HF: Visualisation and interpretation. LJ, SW, KW and CL: Writing - reviewing and editing. HF: Supervision. All authors contributed to the article and approved the finalised version.

## Glossary

**Table:**

ABC	ATP-binding cassette
ACC	Acetyl-CoA carboxylase
ACLY	ATP-citrate lyase
ADC	Adenocarcinoma
AFADESI-MSI	Airflow-assisted desorption electrospray ionisation mass spectrometry imaging
ATP	Adenosine triphosphate
CA19-9	Carbohydrate antigen 19-9
CSCs	Cancer stem cells
ctDNA	Circulating tumor DNA
CTLA-4	Cytotoxic T lymphocyte-associated antigen-4
DESI	Desorption electrospray ionisation
DESI-MSI	Desorption electrospray ionisation mass spectrometry imaging
DLBCL	Diffuse large B-cell lymphoma
FASN	Fatty acid synthase
GLUTs	Glucose transporters
GSH	Glutathione
HCC	Hepatocellular carcinoma
HSF1	Heat shock factor
IDH	Isocitrate dehydrogenase
LC-MS	Liquid chromatography-mass spectrometry
MALDI	Matrix-assisted laser desorption/ionisation
MALDI-MS	Matrix-assisted laser desorption/ ionisation mass spectrometry
MALDI-MSI	Matrix-assisted laser desorption/ionisation mass spectrometry imaging
MALDI-TOF-MSI	Matrix-assisted Laser desorption/ionisation time of flight mass spectrometry
MDSCs	Myeloid-derived suppressor cells
MSI	Mass spectrometry imaging
NADPH	Nicotinamide adenine dinucleotide phosphate
NMR	Nuclear magnetic resonance
NSCLC	Non-small cell lung cancer
OXPHOS	Oxidative phosphorylation
PD-1	Programmed cell death 1
PD-L1	Programmed cell death-ligand 1
ROS	Reactive oxygen species
SAM	S-adenosylmethionine
SIMS	Secondary ion mass spectrometry
SqCC	squamous cell carcinoma
TAMs	Tumor-associated macrophages
TCA	Tricarboxylic acid
TNBC	Triple-negative breast cancer
